# Red Light Phototherapy Using Light-Emitting Diodes Inhibits Melanoma Proliferation and Alters Tumor Microenvironments

**DOI:** 10.3389/fonc.2022.928484

**Published:** 2022-06-24

**Authors:** Evan Austin, Alisen Huang, Jennifer Y. Wang, Marc Cohen, Edward Heilman, Emanual Maverakis, Josef Michl, Jared Jagdeo

**Affiliations:** ^1^Department of Dermatology, State University of New York (SUNY) Downstate Medical Center, Brooklyn, NY, United States; ^2^Department of Dermatology, University of California (UC) Davis Medical Center, Sacramento, CA, United States; ^3^Department of Pathology, SUNY Downstate Medical Center, Brooklyn, NY, United States

**Keywords:** melanoma, phototherapy, low level light therapy, photobiomodulation therapy, reactive oxygen species, tumor micro environment

## Abstract

**Background:**

Total annual cancer rates have decreased due to improved treatment and prevention. However, the incidence of melanoma is rising, and not all patients respond to immune and targeted approaches. Therefore, we sought to determine the efficacy of red light (RL) phototherapy in preclinical models of melanoma.

**Methods:**

Melanoma cells (A375, B16F10, MNT-1) were irradiated with RL. Melanoma proliferation, apoptosis, oxidative stress, and p53 phosphorylation were measured *in vitro*. In C57BL/6 mice, phototherapy safety, B16F10 tumor growth, and immunocyte infiltration were assessed following RL.

**Results:**

*In vitro*, 640 J/cm^2^ RL decreased cellular proliferation without increasing apoptosis, while 1280 J/cm^2^ increased apoptosis. RL increased intracellular reactive oxygen species generation and p53 phosphorylation. In animal models, 2560 J/cm^2^ RL significantly prevented melanoma growth and increased the expression of CD103+ dendritic cells. 1280 and 1920 J/cm^2^ RL decreased tumor volume, but not significantly. RL did not cause skin inflammation or erythema in normal skin.

**Conclusion:**

RL represents a potentially safe and effective melanoma therapeutic. RL prevented tumor growth and increased the expression of immune markers, such as CD103, that are associated with favorable melanoma outcomes. Further research is needed to determine the optimal clinical treatment regimen for melanoma using RL.

## Introduction

While overall yearly cancer rates have decreased due to improved identification and prevention, the incidence of melanoma is increasing (1–3). For primary melanomas (Stage I and II), surgery is usually recommended with margins up to 2 cm (4, 5). Treatments for metastatic melanoma include immune modulators, kinase (i.e., targeted) inhibitors, and radiation therapy (5). Immune checkpoint inhibitors and kinase inhibitors may improve overall survival in up to 50% of patients (6). Despite improvements in outcomes, patients may have or develop resistance to kinase and immune checkpoint inhibitors (5). To improve efficacy, immune checkpoint inhibitors have been used in combination with radiation therapy *via* abscopal effects (7, 8).

Red light (RL) phototherapy may also be a beneficial adjunctive melanoma therapy by inhibiting tumor growth and augmenting anti-tumor immune activity. RL may non-thermally alter cellular biology *via* a process termed photobiomodulation (9). RL absorption by cytochrome c oxidase, a photoreceptive chromophore, excites electrons, activates the electron transport chain, and generates second messengers such as reactive oxygen species (ROS) (9, 10). Cancers, including melanoma, have dysregulated ROS homeostasis and may be particularly sensitive to oxidative stress (9, 11). Increasing intracellular ROS in cancer cells may lead to cell death or inhibition of growth and metastasis (12–14). Herein, we assessed the efficacy of RL phototherapy as a treatment for melanoma using translational models. *In vitro*, RL decreased melanoma cell proliferation and increased apoptosis, oxidative stress, and p53 phosphorylation. In mouse models, RL decreased melanoma tumor growth and increased the association of immune markers related to beneficial tumor immune microenvironments.

## Materials and Methods

### Cell Culture

Monolayers of A375 (ATCC), B16F10 (ATCC), and MNT-1 (ATCC) melanoma cells were cultured in DMEM (Thermo Fisher) with 10% fetal bovine serum (FBS) and 1% Antibiotic-antimycotic mixture (ABAM). Cell cultures were maintained in a humidified incubator at 37°C with 5% carbon dioxide and atmospheric oxygen. For experiments, cells were seeded at low confluency (4,000 cells per 1.77 cm^2^ surface area) (15). Twenty-four hours after plating, melanoma cells were irradiated with RL. **Supplemental Table 1** includes descriptions of the A375, MNT-1, and B16F10 cell lines.

### *In Vitro* Irradiation

Melanoma cells were irradiated with an RL array (633 ± 15-nm, 87 mW/cm^2^ at the light-emitting diode (LED) array surface, Omnilux Revive 2) at 640 and 1280 J/cm^2^. Cells receiving 640 and 1280 J/cm^2^ RL were irradiated outside the incubator for 2 and 4 hours (media temperature 34°C). Each RL-treated group was compared to a temperature, humidity, and CO_2_ matched control group on a heating block (34°C). The time-matched controls for 640 and 1280 J/cm^2^ are 2H (2 hours) and 4H (4 hours) controls, respectively. Experimental irradiations were performed with DMEM (Thermo Fisher) supplemented with FBS and ABAM but without phenol red. Phenol red may alter antioxidant capacity, and thereby confound experiments (16). Additionally, A375 cells were cultured in CO_2_-independent media to assess the effect of media pH on our findings (**Supplemental Methods**). Similar findings were observed when A375 cells were cultured in DMEM or CO_2_-independent media, suggesting the results were not due to environmental conditions during treatment (**Supplemental Figure 1**).

### Crystal Violet Cell Count

Cell counts were assessed using crystal violet (Thermo Fisher) (17, 18). Following treatment with RL, experimental and control samples were incubated for 48 hours to allow cell growth. Collected cells were fixed with 4% formaldehyde (Thermo Fisher) and stained with 0.1% crystal violet. Crystal violet was eluted with 10% acetic acid (Sigma), and optical density (OD) was quantified with a Biotek plate reader at 590-nm. Relative cell count was determined by comparing the OD of the RL and control samples.

### Trypan Blue Cell Count and Viability

48 hours after RL irradiation, cell counts were assessed with a hemocytometer, according to previous protocols (19).

### Cell Cycle Analysis

Cell cycle distribution was assayed using propidium iodide flow cytometry according to previously published protocols (20, 21). 24 hours after plating, A375 and MNT-1 cells were serum-starved (0% FBS) for 24 hours, then irradiated with RL. Fluorescent intensity was then immediately analyzed with flow cytometry (BD Fortessa). Cell cycle distribution was determined with Flowjo software using the Watson pragmatic algorithm (BD Biosciences).

### Apoptosis/Necrosis

Annexin-V and 7-aminoactinomycin D (7-AAD; Sigma Millipore) were used to assay apoptosis/necrosis 24 hours post-RL irradiation using flow cytometry according to the manufacturer’s recommendation (20, 22). Gating was assessed using a positive control sample heated at 70°C for 10 mins before annexin-v and 7-AAD staining. *Post-hoc* gating and analysis were performed with Flowjo software.

### Free Radical Reactive Oxygen Species (ROS) Generation

Melanoma cells were assayed using DHR-123 at 0 hours post-RL irradiation (23). Cells were irradiated with RL and then stained with 1 ml of 1:100 DHR-123 (Thermo Fisher). Non-fluorescent DHR-123 converts to fluorescent rhodamine-123 in the presence of ROS. Rhodamine-123 median fluorescent intensity (MFI) was quantified with flow cytometry. *Post-hoc* gating and analysis were performed with Flowjo software.

### DNA Damage

DNA from cells was collected (Qiagen FlexiGene DNA isolation kit) at 0 or 3 hours following RL or 10 J/cm^2^ of UVB (positive control) irradiation. DNA was then collected according to the manufacturer’s recommendation. DNA quantity and quality were measured with a Nanodrop (Thermo Fisher). CPDs were quantified using ELISA according to the manufacturer’s protocols (Cellbiolabs) with a plate reader at 405-nm.

### Antioxidant Pretreatments

Cells were pretreated with 0.25 to 7.5 mM NAC (Sigma), a free radical scavenger, in DMEM for 2 hours to assess ROS-mediated pathways. Following antioxidant treatment, the cells were washed and fresh culture media was added. The cells were then irradiated with 640 J/cm^2^ RL. 48 hours following RL treatment, changes in cell proliferation were assessed using crystal violet as described above.

### Protein Collection and Western Blot

Protein was collected from RL treated and control cells 24 hours post-irradiation. Western blot was performed according to the manufacturer’s protocols (**Supplemental Methods**).

### Mouse Care and Use

C57BL/6 mice were housed and cared for in the animal facility in the Department of Comparative Medicine at SUNY Downstate (IACUC ID: 19-10564). Animals were provided a standard chow diet and always had full access to food and water.

### Mouse Irradiations

Female C57BL/6 mice were irradiated daily with RL phototherapy (633 ± 15-nm, Omnilux Revive 2) at a power density of 87 mW/cm^2^. Mice were treated in custom-designed temperature-controlled treatment cages to prevent supraphysiologic temperatures from the RL array and light (**Supplemental Figure 2**). Daily treatment regimens were 1280, 1920, and 2560 J/cm^2^ which corresponded to 4 hours (4H), 6 hours (6H), and 8 hours (8H) of RL treatment, respectively. The ambient temperature in the cages was maintained using a temperature probe in the cage and air-conditioning unit. Mouse core body temperature was maintained between 34.5 and 38.9°C measured using a rectal temperature probe.

#### Safety Regimen

On day 0, the backs of the female C57BL/6 mice were shaved and naired while anesthetized with inhaled isoflurane. On day 1, 3 mice were randomly allocated to each control or RL treatment (daily 1280, 1920, or 2560 J/cm^2^) group (n=3). On days 1-15, the mice received daily irradiations of 1280, 1920, or 2560 J/cm^2^. During daily RL treatments, the mice were observed for changes in behavior, and rectal temperatures were recorded. On day 16, the mice were euthanized. Skin sections were fixed in 10% formalin and processed for IHC.

#### Efficacy Regimen

RL efficacy was assessed in female C57BL/6 mice injected with 3 x 10^5^ B16F10 cells. Starting on day 3, the mice received daily irradiations of 1280 (n=10), 1920 (n=10), or 2560 J/cm^2^ (n=12). To prevent overcrowding during the treatment protocol, the 1280, 1920, and 2560 J/cm^2^ treatment regimens were performed separately with an equal number of control mice (n=10 for 4H control, n=10 for 6H control, and n=12 for 8H control). To prevent overcrowding during the treatment protocol the 1280, 1920, and 2560 J/cm^2^ treatments were performed separately with an equal number of control mice (n=10 for 4H control, n=10 for 6H control, and n=12 for 8H control). The mice and tumors were assessed for humane outcomes (e.g., tumor rupturing, bleeding, immobilization) before, during, and after RL treatments. Daily irradiations continued until a single mouse required euthanization for a humane endpoint, at which point, all control and RL-treated mice were euthanized (day 13 for 1280 and 1920 J/cm^2^, day 15 for 2560 J/cm^2^). Mice and melanoma tumor dimensions were tracked daily and photographed with a Nikon D3500 following euthanization.

### Quantification of Melanoma Growth

Tumors were excised from euthanized mice and measured in three dimensions to confirm the calculated volume. The overlying skin was preserved *in situ* for histologic analysis. Tumor volume was calculated from the excised tumor using the following formula that includes a depth parameter (24):


$$Volume=\frac{\pi }{6}Lenght\times Width\times Depth$$

### Tissue Histology

Following euthanization, tumors were excised and fixed in 10% formalin. The fixed skin and tumor samples with *in situ* skin were sent to Histowiz (Brooklyn, NY) for processing according to a standard operating procedure and fully automated workflow (**Supplemental Methods**).

### Quantification of IHC Staining Intensity

Whole tumor and skin section images were imported into the HALO software database (Indica Labs). Quantitative biomarkers were analyzed using Multiplex IHC, area quantification, and tissue classifier modules. IHC staining was indexed to the viable tumor or skin area for all analyses. A dermatopathologist (EH) confirmed the validity of immunohistochemical staining.

### Data Analysis

Data analysis was performed for all paired assays (RL to matched controls) using a two-tailed T-test. Analysis of Variance (ANOVA) was used to compare experiments with multiple comparison groups. Statistical significance was determined with a P-value of less than 0.05. GraphPad software was used for statistical testing and figure generation.

## Results

### RL Phototherapy of Melanoma Decreases Cell Count by Inhibiting Proliferation and Increasing Cell Apoptosis

**Figure 1:**
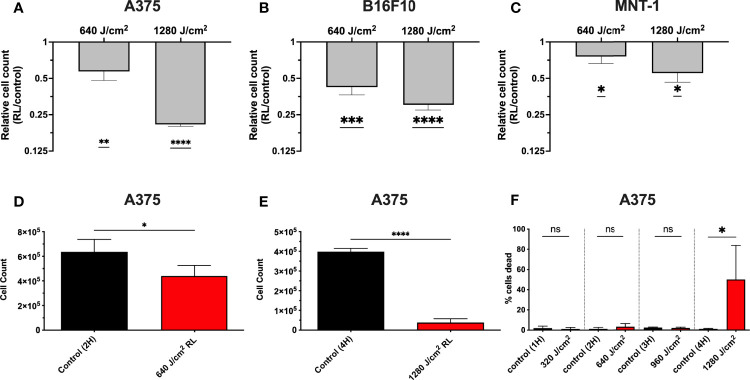
RL causes a dose-dependent decrease in melanoma cell counts. **(A)** 48 hours after RL irradiation, relative cell counts were assessed in A375 (n=4), **(B)** B16F10 (n=5), and **(C)** MNT-1 (n=4) cells using crystal violet. The crystal violet was eluted and quantified using a Biotek plate reader, with an OD reading of 590-nm. The RL group’s 590-nm OD was indexed to matched-control for graphing of relative cell count. **(D)** Cell count was measured using a hemocytometer in A375 48 hours after treatment with 640 (n=4) and **(E)** 1280 J/cm^2^ (n=4). **(F)** Cell viability was measured using a hemocytometer 48 hours after treatment with 640 (n=4) and 1280 J/cm^2^ (n=4). A two-tailed T-Test (p<0.05) compared OD, cell count, and cell viability of RL treated cells to time-matched control. OD, optical density; RL, red light; 2H, 2 hours; 4H, 4 hours.*denotes p<0.05, ** denotes p<0.01, *** denotes p<0.01, **** denotes p<0.0001, and ns denotes not significant.

We have demonstrated that 320 and 640 J/cm^2^ RL decreases human dermal fibroblasts (HDFs) proliferation and modulates the expression of fibrotic and oxidative stress pathways (19, 25, 26). Other researchers have found that blue light, but not RL at fluences up to 360 J/cm^2^, decreased B16F10 melanoma survival and proliferation (27–29). Low fluence RL has had variable effects (i.e., no changes, increases, or decreases) on survival in other cancer models (e.g., lung cancer, squamous cell carcinoma, glioblastoma, and breast cancer) (29–31). Thus, unpigmented (A375) and pigmented (MNT-1 and B16F10) melanoma cells were irradiated with 640 and 1280 J/cm^2^ RL to determine whether higher fluences may achieve therapeutic outcomes. At 48 hours following irradiation, there was a dose-dependent decrease in A375, MNT-1, and B16F10 cell counts as measured by crystal violet staining intensity (**Figures 1A–C**). The results were confirmed using a hemocytometer at 48 hours in A375 cells treated with 640 and 1280 J/cm^2^ (**Figures 1D,E**). At 48 hours, 1280 J/cm^2^ significantly increased cell death, while 640 J/cm^2^ RL did not (**Figure 1F**).

**Figure 2:**
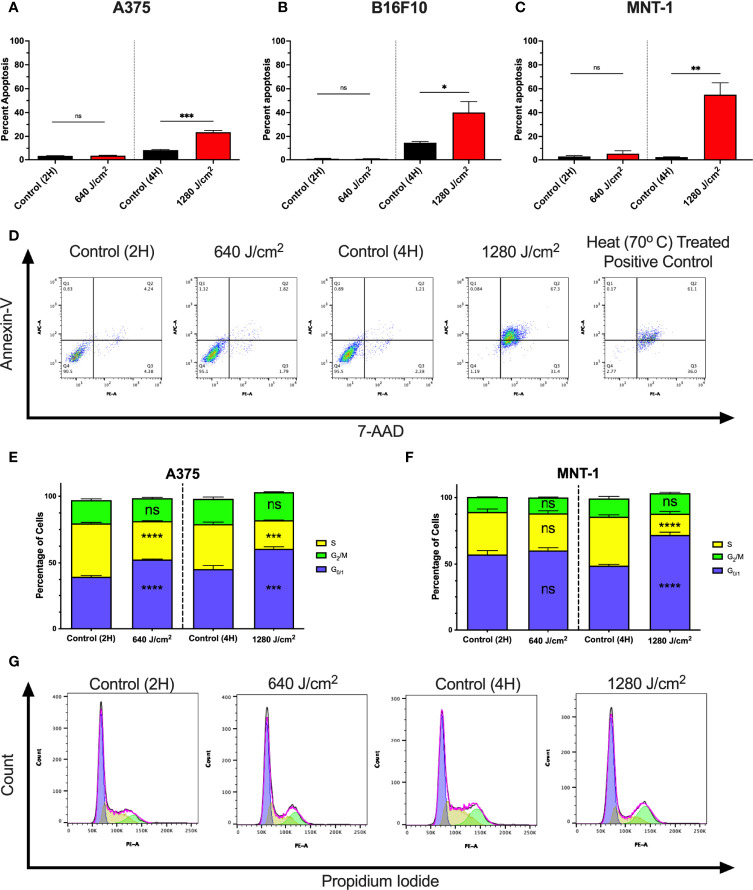
RL increases cell death and alters the cell cycle. **(A)** Cell death was confirmed in A375 (n=3), **(B)** B16F10 (n=5), and **(C)** MNT-1 (n=5) melanoma cells using annexin-v and 7-AAD flow cytometry. **(D)** Representative flow plots for annexin-V and 7-AAD. A heat-treated positive control (70°C) was used for gating. **(E)** Cell cycle progression was assessed using PI flow cytometry in A375 (n=3) and **(F)** MNT-1 (n=3) cells. The percentage of cells in each phase was modeled using the Watson pragmatic model in FlowJo. **(G)** Representative cell cycle phase histograms from the A375 cell line. Apoptosis and cell cycle distribution compared RL and time-matched control groups using a two-tailed T-Test (p<0.05). 2H, 2 hours; 4H, 4 hours; PI, propidium iodide; 7-AAD, 7-Aminoactinomycin D; and RL, red light. *denotes p<0.05, ** denotes p<0.01, *** denotes p<0.001, **** denotes p<0.0001, and ns denotes ns.

To determine whether increases in cell death were due to apoptosis, A375, MNT-1, and B16F10 cells were irradiated with RL and analyzed using annexin-V and 7-Aminoactinomycin D (7-AAD) flow cytometry (**Figures 2A–C**) (32, 33). At 24 hours post-irradiation, 1280 J/cm^2^ (but not 640 J/cm^2^) resulted in a significant increase in apoptosis compared to control. Representative annexin-V and 7-AAD flow plots are shown in **Figure 2D**.

We tested whether RL regulates the cell cycle as 640 J/cm^2^ decreased cell count without increasing cell death. At 24 hours post-irradiation, 640 and 1280 J/cm^2^ RL significantly decreased the percentage of A375 cells in S-phase and increased the percentage in G_0/1_ (**Figures 2D**). In MNT-1 cells, 1280 J/cm^2^ significantly decreased the percentage of cells in the S-phase and increased the percentage in the G_0/1_-phase (**Figure 2E**). MNT-1 cells irradiated with 640 J/cm^2^ had decreased, but not significant, alterations in S or G_0/1_ (**Figure 2F**). Representative PI flow plots are shown in **Figure 2G**.

### Regulation of the Cell Cycle and Apoptosis Was Associated With Increased p53 Phosphorylation

**Figure 3:**
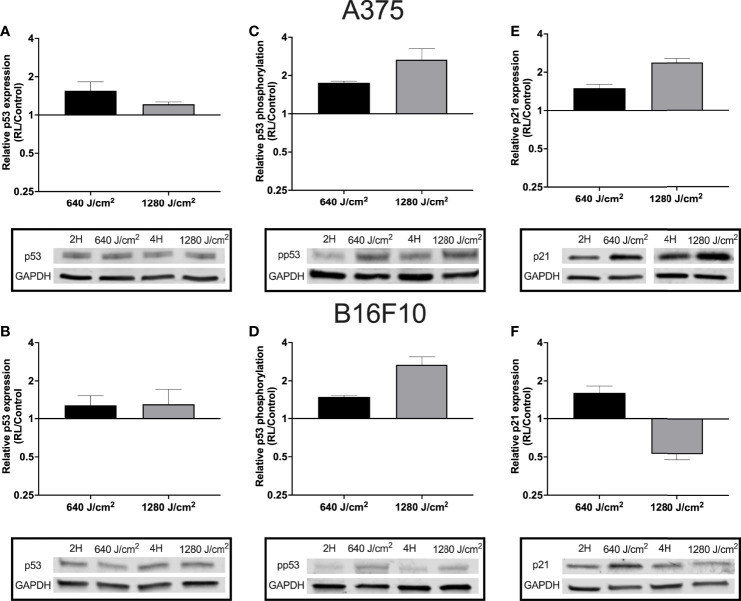
RL increases p53 expression and phosphorylation. Protein was collected from A375 and B16F10 cells at 24 hours following irradiations with 640 and 1280 J/cm^2^. Relative expression of p53 in **(A)** A375 and **(B)** B16F10. Isolated protein from A375 cells treated with 640 and 1280 J/cm2 RL or control was probed with p53 antibodies twice (n=2) and six times (n=6), respectively. Isolated protein from B16F10 cells treated with 640 and 1280 J/cm2 RL or control was probed with p53 antibodies three times (n=3) and twice (n=*2*). **(C)** p53 phosphorylation was measured in A375 and **(D)** B16F10 cells. In A375 cells, 640 and 1280 J/cm^2^ treated samples were run five-times (n=5) and three times (n=3), respectively. In B16F10 cells, 640 and 1280 J/cm^2^ treated samples were analyzed with phosphorylated p53 antibodies twice (n=2) and five times (n=5). **(E)** p21 expression in A375 and **(F)** B16F10 cells following irradiation with 640 and 1280 J/cm^2^ RL. In A375 cells, 640 and 1280 J/cm^2^ treated samples were run five-times (n=5) and twice (n=2). In B16F10 cells, 640 and 1280 J/cm^2^ treated samples were run three-times (n=3) and twice (n=2). 2H, 2 hour control; 4H, 4 hour control; 640, 640 J/cm^2^; 1280, 1280 J/cm^2^; ns, not significant; and RL, Red light.

As RL may increase cell apoptosis and regulate the cell cycle, p53 expression and phosphorylation were measured in B16F10 and A375 cells using western blot. Phosphorylation of p53 at Ser15 promotes the dissociation of p53 from MDM2 (HDM2), apoptosis activation, and cell cycle regulation (34, 35). B16F10 and A375 were examined as these cell lines are wild-type for p53 (36). Protein from A375 and B16F10 cells was collected 24 hours following irradiation. In A375 cells, 640 and 1280 J/cm^2^ increased p53 by 1.2 to 1.6 fold (**Figure 3A**). In B16F10 cells, 640 and 1280 J/cm2 RL both increased total p53 by 1.3-fold (**Figures 3B**). Phosphorylated p53 increased by 1.8-fold and 2.7-fold in 640 and 1280 J/cm^2^ treated A375 cells and by 1.5-fold and 2.7-fold in 640 and 1280 J/cm^2^ treated B16F10 cells (**Figures 3C, D**).

Next, p21 expression was measured as p53 phosphorylation increases the transcription of *CDKN1*, the gene for p21 (an inhibitor of the cell cycle progression) (34, 37). In A375 cells, there was a dose-dependent increase in p21 expression following RL irradiation (**Figure 3E**). However, in B16F10 cells, p21 expression increased following 640 J/cm^2^ RL but decreased after 1280 J/cm^2^ RL (**Figure 3F**). Knockdown of p21 with siRNA transfection did not restore the cell count of 640 J/cm^2^ RL treated A375 cells (**Supplemental Figure 3**). As a result, p21 knockdown is unlikely to be solely responsible for RL-mediated anti-proliferative effects.

### RL Phototherapy Increases ROS Generation but Does Not Induce the Formation of Cyclobutane Pyrimidine Dimers (CPDs)

**Figure 4:**
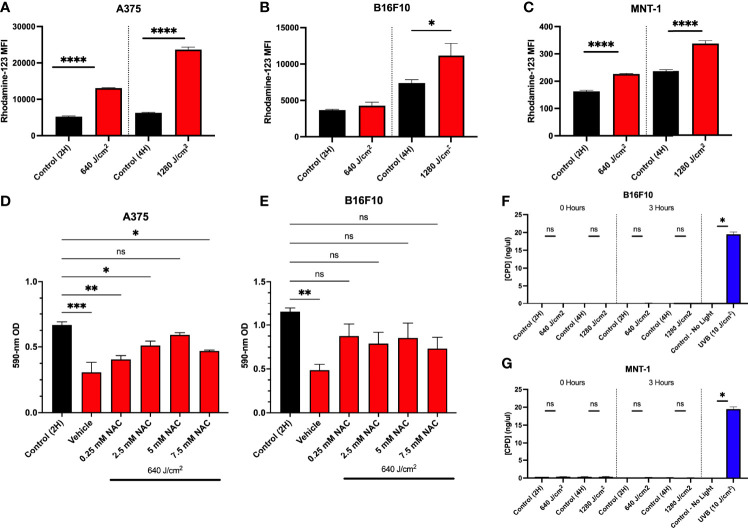
Increases in ROS due to RL decrease cell proliferation. **(A)** A375 (n=3), **(B)** B16F10 (n=5), and **(C)** MNT-1 (n=4) cells were irradiated with 640 and 1280 J/cm^2^ RL, and rhodamine-123 MFI was assessed at 0 hours post-treatment using flow cytometry. **(D)** A375 (n=4) and **(E)** B16F10 (n=5) cells were pretreated with 0.25 to 7.5 mM NAC and irradiated with 640 J/cm^2^ RL. Cell count was assessed by measuring cell staining intensity with crystal violet 48 hours after irradiation. **(G)** B16F10 and **(F)** MNT-1 cells were irradiated with 640 to 1280 J/cm^2^ RL or UVB (10 J/cm^2^). DNA was collected at 0 and 3 hours post-irradiation and analyzed for CPDs using ELISA. RL and control MFI and CPD concentration for 640 and 1280 J/cm^2^ RL was compared using a two-tailed T-test (p<0.05). ANOVA was used to compare RL and NAC treated groups to control. Cell count was assessed by measuring cell staining intensity with crystal violet 48 hours after NAC. Ordinary one-way ANOVA (p<0.05) compared NAC pretreatments and control. Dunnett’s *post-hoc* testing compared the mean of every RL group to control. 2H, 2 hour; 4H, 4 hour; UVB, ultraviolet B; NAC, n-acetylcysteine; OD, optical density. *denotes p<0.05, ** denotes p<0.01, *** denotes p<0.001, **** denotes p<0.0001, ns and denotes not significant.

p53 may be activated by cellular stress such as DNA damage or directly by ROS. Photobiomodulation increases ROS production *via* activation of electron transport and mitochondrial dysfunction (9, 38). We have previously demonstrated that 640 J/cm^2^ RL increased intracellular ROS in HDFs (19). Immediately after 640 and 1280 J/cm^2^ RL (0 hours), the cells were treated with dihydrorhodamine-123 (DHR-123) to measure intracellular ROS. 640 and 1280 J/cm^2^ increased intracellular ROS in A375 and MNT-1 (**Figures 4A, C**). In B16F10 cells, 1280 J/cm^2^ RL increases intracellular ROS production (**Figure 4B**).

To confirm whether increases in ROS were mechanistically linked to decreases in cell proliferation, we pretreated A375 and B16F10 cells with an antioxidant, n-acetylcysteine (NAC), and irradiated cells with 640 J/cm^2^ RL. NAC sequesters ROS and has previously been used in melanoma research to study oxidative stress (12, 39). In A375, pretreatment with 5 mM NAC prevented 640 J/cm^2^ RL mediated decreases in cell count (**Figure 4D**). In B16F10 cells, 0.25 mM to 7.5 mM NAC prevented decreases in cell count following RL phototherapy (**Figure 4E**).

ROS from 60 J/cm^2^ of UVA has been shown to interact with melanin and induce CPDs within 3-hours post-irradiation *via* chemiexcitation (40). Others have found that 5 J/cm^2^ RL did not cause CPDs in MNT-1 and B16F10 cells (41). CPDs in DNA were measured from RL irradiated MNT-1 and B16F10 cells (**Figures 4F, G**). Neither 640 nor 1280 J/cm^2^ RL induced DNA damage by 3 hours post-irradiation.

### RL at Fluences up to 2560 J/cm^2^ Is Safe in Mice

**Figure 5:**
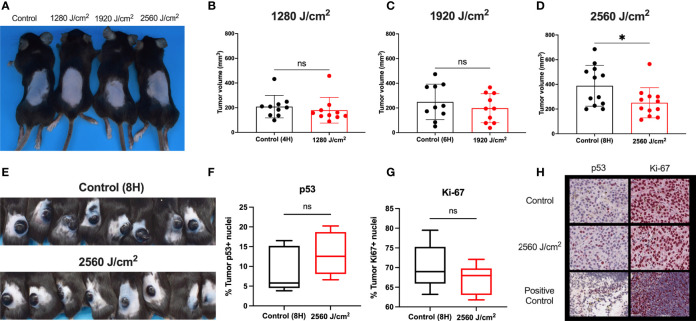
RL safety and efficacy. **(A)** C57BL/6 mice without tumors were treated with RL at 1280, 1920, and 2560 J/cm^2^ for 15 days (n=3). Mice had no increase in rectal temperature, and the skin was non-inflamed and non-erythematous compared to non-RL treated mice. **(B)** C57BL/6 Mice were injected with 3 x 10^5^ melanoma cells and irradiated daily with 1280 J/cm^2^ (n=10), **(C)** 1920 J/cm^2^ (n=10), and **(D)** 2650 J/cm^2^ RL (n=12). Volume was calculated using the formula Volume = 0.52 x length x width x depth. **(E)** Representative mice with tumors (n=8) in the control group and 2560 J/cm^2^ RL group on day 15. **(F)** Quantification of p53+ (n=5) and **(G)** Ki-67+ (n=5) staining nuclei in from control (8H) and 2560 J/cm^2^ treated tumors. Quantification of staining was performed using Indica HALO software. **(H)** Representative images of p53+ and Ki-67+ staining in tumors. Cephalic (embryonic day 14) and spleen positive control sections are provided for p53 and Ki-67. Excised tumor volumes and IHC staining intensity for RL-treated mice were compared to matched controls by a two-tailed T-test (p<0.05). *denotes p<0.05. ns denotes not significant.

To translate *in vitro* findings to clinical regimens, the safety of RL phototherapy was first assessed in normal C57BL/6 mice (i.e., without melanoma inoculation). Daily 1280, 1920, and 2560 J/cm^2^ RL sessions were administered. The mice were awake and unrestrained during RL irradiations to prevent stress-related immunosuppression (42, 43). Higher fluences were tested *in vivo* as mice often require higher therapeutic drug doses than *in vitro* cell culture and human patients due to differences in body physiology and metabolism (44). Interspecies allometric dosing conversion equations exist for pharmaceuticals but are not available for phototherapeutic interventions (44). After 15 days of treatment, the RL-treated mice had no increase in rectal temperature, and the skin was non-inflamed and non-erythematous compared to non-treated mice (**Figure 5A**).

### RL Phototherapy Inhibited Melanoma Tumor Growth *In Vivo*

As RL was safe in normal mouse skin, C57BL/6 mice were intradermally injected with 3x10^5^ B16F10 cells to investigate the efficacy of RL phototherapy for melanoma (45). On day three following melanoma inoculation, the mice were randomly sorted into RL treated and control groups (45). There was no difference in tumor surface area between the RL and control groups before initiating treatment (**Supplemental Figure 4**). Mice were then irradiated with 1280, 1920, and 2560 J/cm^2^ daily until humane endpoints (i.e., the tumor bled or ruptured) (45). RL caused a dose-dependent decrease in tumor volume (**Figures 5B–D**). In the 2560 J/cm^2^ RL-treated group, tumor volumes were significantly smaller than the control (p<0.05, **Figure 5D**). Figure E shows intact tumors on the backs of 8 control and 2560 J/cm^2^ RL-treated mice. Immunohistochemistry (IHC) demonstrated non-significant decreases in Ki-67+(p=0.19, n=8) and increases in p53+ (p=0.28, n=5) in the 2560 J/cm^2^ RL group compared to the control (**Figures 5F–H**).

### RL Increases the Infiltration of CD103+ Dendritic Cells in the Peritumoral Skin

As the C57BL/6 mice used in these experiments were immunocompetent, we assessed the effects of RL phototherapy on dermal and tumor immune infiltration. Tumor immune microenvironment can substantially affect patient prognosis (46–49). IHC was performed with excised tumors and peritumoral skin using immune markers for lymphocytes (CD3, CD4, CD8, FoxP3), dendritic cells (CD103), macrophages (CD68), and neutrophils (Ly6G).

**Figure 6:**
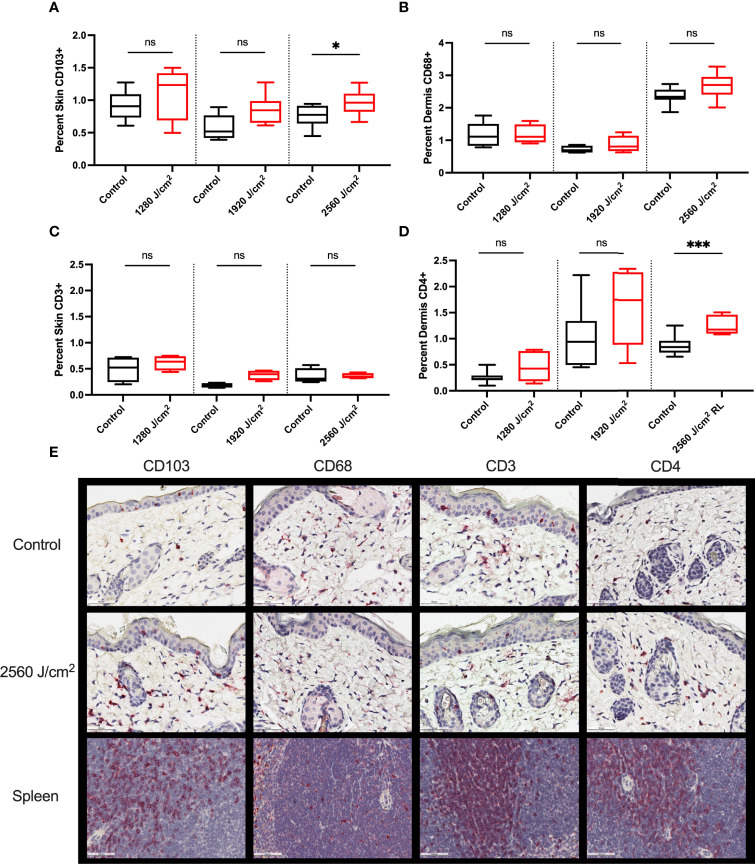
RL increases the expression of immunocyte markers. **(A)** Quantification of CD103 (n=8), **(B)** CD68 (n=5 for 1280 J/cm^2^, n=4 for 1920 J/cm^2^, n=8 for 2560 J/cm^2^), **(C)** CD3 (n=4), and **(D)** CD4 (n=8) staining in the peritumoral dermis or skin from 1280, 1920, and 2560 J/cm^2^ RL-treated and control C57BL/6 mice injected with B16F10 cells (two-tailed T-Test, p<0.05). **(E)** Representative IHC for CD103, CD68, CD3, and CD4 positive staining peritumoral dermis and skin. The staining intensity of each marker was indexed to the total skin or dermal area for each section. Spleen positive control tissue is provided. Staining was quantified using Indica HALO software and compared to total skin or dermal area. * denotes p<0.05, *** denotes p<0.001, ns denotes not significant.

Control and RL-treated tumors were negligibly stained for all immune markers, suggesting the exclusion of immune cells from the tumor (data not shown). The peritumor skin stained positively for CD103, CD68, CD3, and CD4 (**Figure 6**) but negligibly for CD8, Foxp3, and Ly6G (data not shown). 2560 J/cm^2^ RL significantly increased CD103+ expression in peritumoral skin (**Figure 6A**). 2560 J/cm^2^ RL increased CD68+ dermal staining, but not significantly (**Figure 6B**). CD4, but not CD3, expression increased in response to RL phototherapy (**Figures 6C, D**). IHC for CD103, CD68, CD3, and CD4 was also assessed in non-tumor mice to determine the effects of RL on immune function without cancer (**Supplemental Figure 5**). RL caused a significant decrease in CD103 expression at 1280-1920 J/cm^2^ and a modest dose-dependent increase in CD4+ expression in normal mouse skin.

## Discussion

Herein, we investigated the anti-tumor effects of RL phototherapy using *in vitro* and vivo models of melanoma. The ultimate goal of our RL preclinical experiments was to demonstrate therapeutic proof-of-concept and identify underlying mechanisms of action.

*In vitro*, 640 and 1280 J/cm^2^ RL phototherapy decreased cell proliferation, while 1280 J/cm^2^ RL also increased cell apoptosis. Cell cycle regulation and apoptosis were associated with p53 activation and increased ROS generation. Activating p53 may be a beneficial mechanism as approximately 80-90% of melanomas are wild-type for p53 (50, 51). Wild-type p53 can regulate cell cycle checkpoint progress and induce apoptosis in response to oxidative stress or DNA damage repair pathways (52, 53). In HDFs, 640 J/cm^2^ similarly did not increase p21 expression, despite increasing p53 phosphorylation (54). Additionally, suprathreshold oxidative stress has been shown to inhibit melanoma growth and promote apoptosis independently (14). Pretreatment of melanoma cells with NAC inhibited the anti-proliferative effects of RL phototherapy in melanoma cells. Others have similarly found that antioxidants, including NAC, increased melanoma metastasis in mouse models (12, 39). Therefore, increased oxidative stress may be a therapeutically beneficial mechanism of action (55).

We then translated RL phototherapy protocols to animal models after demonstrating anti-tumor effects *in vitro*. B16F10 melanoma cells were used for *in vivo* protocols as the tumors grow rapidly and with consistent kinetics (45, 56). As such, multiple RL treatment fluences (i.e., 1280, 1920, and 2560 J/cm^2^) could be screened for potential efficacy. Additionally, C57BL/6 mice injected with B16F10 are immunocompetent, thus allowing for the assessment of immune function. In mice, irradiation of 2560 J/cm^2^ for up to 13 days decreased B16F10 melanoma tumor size compared to control. These results differ from previous studies in which three days of 1050 J/cm^2^ RL from an In-Ga-Al-AsP laser (660-nm) increased murine melanoma tumor growth (57). As a result, higher fluences of RL and longitudinal treatment protocols may be necessary for clinical translation. Future clinical trials are necessary to convert RL phototherapy dosing regimens from cell culture and mice to patients and evaluate the efficacy of different phototherapy regimens, including photobiomodulation, photothermal therapy, and photodynamic therapy (58, 59).

To determine whether RL may be combined with existing pharmaceutical approaches, including immune checkpoint inhibitors, we investigated the effects of RL phototherapy on tumor immune responses. RL increased the expression of CD103+, a marker of migratory dendritic cells that enhance antigen presentation to cytotoxic T-cells in the lymph nodes (60–62). CD103+ expression is linked to favorable immune microenvironments, improved prognoses, prevention of metastasis, and responsiveness to PD-1/PD-1L inhibitor therapy (60–62). CD4 expression, classically a helper T-cell marker, was similarly increased following RL phototherapy. However, CD4 is also non-specifically expressed on macrophages and dendritic cells, and flow cytometry is necessary to fully characterize the immune cell populations and T-cell infiltration (63). Other researchers have also examined the role of immune activity in phototherapy (64, 65). Ottaviani et al. used RL phototherapy to decrease tumor volume and increase the expression of type I interferons (65, 66). Type I interferons are involved with immune surveillance, dendritic cell maturation, and inflammatory responses (65, 66). Together, previous research and our results suggest that RL may activate systemic immune responses in melanoma mouse models and facilitate a therapeutic immune niche. Therefore, RL phototherapy may be synergistically combined with current melanoma therapies for the benefit of patients.

Our research on RL phototherapy has strengths and weaknesses. One strength is that we utilized multiple *in vitro* and *in vivo* protocols to confirm laboratory findings. Another strength is that the culture media and mouse core body temperatures were carefully maintained at physiological levels during irradiations. Controlling temperature is essential as increased heat (40°C and above) may independently lead to decreased cell viability, increased ROS generation, G_1_ cell cycle arrest, membrane denaturation, and coagulative necrosis (15, 67–70). 450 J/cm^2^ RL (650-nm) has been shown to cause membrane protein denaturation in red blood cells (70). Three human studies by our research team tested the safety of LED RL in patients, and fluences of 320-480 J/cm^2^ (treatment duration of 1-1.5 hours) caused occasional erythema and blistering, respectively, in patients without the use of a cooling device (71, 72). In mice, fluences up to 2560 J/cm^2^ of RL with air-conditioning did not induce erythema, blistering, or ulceration in non-tumor mouse skin. Cooling devices are often incorporated in laser systems, and future clinical trials could test higher fluences with temperature regulation (73). However, photothermal reactions and inefficient energy transfer may increase intracellular temperature despite external cooling (15, 68, 70). A potential weakness of our mouse protocols is that the mice were awake and unrestrained to prevent immunosuppression. Therefore, the mice were able to huddle up and turn away from the light, which resulted in variability in treatment fluence depending on individual mouse behavior (42, 43). In future experiments, higher power density LEDs or lasers may be tested to reduce total treatment duration.

RL represents a potentially promising approach for melanoma therapy, as RL is inexpensive, noninvasive, easily combined with existing melanoma pharmacologic treatments, and associated with low morbidity and no known mortality. Additionally, RL can be made available for home use to augment existing melanoma therapies under the guidance of a physician. Clinical studies have demonstrated that patients can safely use LED devices at home (74). Clinical translation for melanoma therapy could quickly follow safety and efficacy demonstration in phase I-III clinical trials. We anticipate that soon, it may be possible for patients to use home RL phototherapy to augment therapy for cutaneous melanoma metastasis, empowering patients to participate in their cancer treatment.

## Data Availability Statement

The raw data supporting the conclusions of this article will be made available by the authors, without undue reservation.

## Ethics Statement

The animal study was reviewed and approved by SUNY Downstate IACUC.

## Author Contributions

EA investigated, performed data analysis, data curated, and wrote the original draft. AH investigated and performed data analysis. JW investigated and performed data analysis. MC investigated and performed data analysis. EH performed data analysis and reviewed and edited. JM reviewed and edited and provided resources. EM reviewed and edited and provided resources. JJ reviewed and edited, acquired funding, supervised, performed projected administration, and provided resources. All authors contributed to the article and approved the submitted version.

## Funding

Research funding was received through SUNY Downstate Department of Dermatology and UC Davis Department of Dermatology startup funds.

## Conflict of Interest

JJ is a consultant for GlobalMed Technologies (Omnilux).

The remaining authors declare that the research was conducted in the absence of any commercial or financial relationships that could be construed as a potential conflict of interest.

## Publisher’s Note

All claims expressed in this article are solely those of the authors and do not necessarily represent those of their affiliated organizations, or those of the publisher, the editors and the reviewers. Any product that may be evaluated in this article, or claim that may be made by its manufacturer, is not guaranteed or endorsed by the publisher.

## Abbreviations

ABAM, Antibiotic-antimycotic mixture; ANOVA, Analysis of variance; CPD, Cyclobutane pyrimidine dimer; DHR-123, Dihydrorhodamine-123; FBS, Fetal bovine serum; HDF, Human dermal fibroblasts; IHC, Immunohistochemistry; LED, Light-emitting diode; MFI, Median fluorescence intensity; NAC, N-acetylcysteine; OD, Optical density; RL, Red light; ROS, Reactive oxygen species; 7-AAD, 7-Aminoactinomycin D.
