# Supplementary material for: Red Light Phototherapy Using Light-Emitting Diodes Inhibits Melanoma Proliferation and Alters Tumor Microenvironments
Source: Front Oncol. 2022 Jun 24;12:928484. doi: 10.3389/fonc.2022.928484 (PMC9278815; doi:10.3389/fonc.2022.928484)
Supplement: Supplementary file 1 [file DataSheet_1.docx]

**Supplemental methods**

In vitro irradiations in CO_2_-independent media

A375 cells were irradiated with an RL array (633 ± 15-nm, Omnilux Revive 2) at 640 and 1280 J/cm^2^. The power density at the LED array surface was approximately 87 mW/cm^2^ using a photometer (Thorlabs). Cells receiving 1280 J/cm^2^ RL were irradiated for 4 hours outside the incubator in CO_2_-independent media (Thermo Fisher). Media temperature was maintained at 34°C to prevent photothermal effects. 1280 J/cm^2^ RL-treated group was compared with a time and temperature matched control group maintained at 34°C on a heating block. For the negative control group, A375 cells were cultured in CO_2_-independent media and maintained in the incubator (5% CO_2_, 37°C) throughout the protocol. Cell counts were assessed using crystal violet at 48 hours after treatment. An ANOVA with Tukey’s post hoc analysis was used to compare the means of the 1280 J/cm^2^ RL, matched control (4H), and incubator control.

Protein Collection and Western Blot

Protein was isolated with cell lysis buffer (RIPA Buffer supplemented with 1:200 protease/phosphatase inhibitor and 1:100 PMSF). According to the manufacturer's recommendations, the protein was quantified using a Bradford assay (Bio-Rad). 20 ug of protein sample with Laemmeli buffer (Bio-Rad) was loaded into each well of an SDS-PAGE gel with 1x tris/glycine/SDS buffer (Bio-Rad). The protein was transferred to a PVDF (Bio-Rad) membrane with 1x tris/glycine buffer and 20% methanol. The membranes were stained with GAPDH, p53, phospho-53, p21. 1:10,000 near-infrared fluorescent secondary antibodies were then used. The LICOR Odyssey system quantifies secondary antibody fluorescent intensity (i.e., protein intensity) at 680 and 800-nm. A complete list of primary and secondary antibodies is available in the supplemental methods.

Antibodies for in vitro experiments

The following validated antibodies were used in vitro western blot experiments: Phospho-p53 (Ser15, 1:200, Cell signaling #9284), p53 (1:200, DO-7, Cell signaling #48818), p53 (1:200 D2H9O, Rodent Specific, Cell Signaling #32532), p21 Waf1/Cip1 (1:1000, 12D1, Cell Signaling #2947), p21 Waf1/Cip1 (1:200 Cell signaling #64016), GAPDH (1:10,000 D16H11, XP®, Cell signaling #5174), GAPDH (1:10,000, D4C6R, Cell signaling #9716), IRDye® 800CW Donkey anti-mouse IgG Secondary Antibody (1:10,000 ,LICOR), IRDye® 680 CW Donkey anti-rabbit IgG Secondary Antibody (1:10,000, LICOR), IRDye® 800CW Donkey anti-rabbit IgG Secondary Antibody (1:10,000, LICOR), IRDye® 680 CW Donkey anti-mouse IgG Secondary Antibody (1:10,000, LICOR).

Antibodies for in vivo experiments

The following validated antibodies were used: CD3 (1:100, Abcam 16669), CD4 (1:100, Abcam 183685), CD8 (1:100, Thermo Fisher 13-0808-80), Ki67 (1:800, Abcam 15580), CD103 (ITGAE; 1:1000, Abcam 224202). Ly6G (1:100, Abcam 25377), FoxP3 (1:100, Cell signaling 12653), p53 (1:100, Leica NCL-L-p53-CM5p).

RNA Interference

According to the manufacturer's recommendation, p21 was silenced in A375 cells using siRNA knockdown (Thermo Fisher). On day 1, 25 or 60 pmol of p21 siRNA was combined with lipofectamine RNAiMAX (Thermo Fisher) in Opti-MEM medium (Thermo Fisher) and added to A375 cell culture. At least 2 different siRNA vectors (s415 and s417; Thermo Fisher) and a scrambled control (SC) were tested to limit the potential for off-target results. On day 3, Pilot experiments demonstrate that 25 and 60 pmol siRNA results in greater than 85% knockdown of p21.

On day 2 (i.e., 24 hours following knockdown), A375 cells transfected with 25 pmol of siRNA were replated in 35 mm culture dishes for experiments. On day 3, transfected A375 cells were irradiated with 640 J/cm^2^ RL. On day 5 (48 hours after RL irradiation), cell counts were performed on the irradiated and p21 silenced melanoma cells to determine if p21 knockdown prevented RL-mediated anti-proliferative effects.

Tissue Histology

IHC was performed on a Bond Rx autostainer (Leica Biosystems) with standard protocols with enzyme treatment (1:1000). Tumor sections were stained with a primary antibody of interest. Sections were stained with antibodies to identify T-cells (CD3+, CD8+, and CD4+, and FOXP3+), dendritic cells (CD103+), and macrophages (CD68+).^27-29^ Tumor proliferation was assessed with Ki-67 and p53 antibody staining. Chromogen pink kit was used as the substrate for IHC instead of 3,3'-Diaminobenzidine (DAB), as brown DAB may be confused with melanin. Bond Polymer Refine Detection (Leica Biosystems) was used according to the manufacturer's protocol. After staining, sections were dehydrated and film coverslipped using a TissueTek-Prisma and Coverslipper (Sakura). Whole slide scanning (40x) was performed on an Aperio AT2 (Leica Biosystems). A full list of primary and secondary antibodies is available in the supplement.

**Supplemental Table**

| **Cell line** | A375 | MNT-1 | B16F10 |
| --- | --- | --- | --- |
| **Braf** | Mutant | Mutant | WT |
| **NRAS** | WT | WT | WT |
| **P53** | WT | N/A | WT |
| **Pigmentation** | No | Yes | Yes |
| **Gender** | F | F | N/A |
| **Species** | Human | Human | Murine |
| **Other mutations** |  |  | p16Ink4a and p19Arf |

**Supplemental Table 1: Classification of tumor cell lines and mutations.**

**Supplemental Figures**

**Supplemental Figure 1. RL decreased cell count in A375 cells cultured in CO_2_-Independent media.** 48 hours after 1280 J/cm^2^ RL irradiation, relative cell counts were assessed in A375 cells (cultured in CO_2_-independent media) using crystal violet. The crystal violet was eluted and quantified using a Biotek plate reader, with an optical density reading of 590-nm. The RL treated and 4H control group were compared to a negative control group in which cell maintained in the incubator throughout the protocol. RL-treated, matched control, and incubator maintained (negative control) groups were cultured in CO_2_ independent media (Thermo Fisher). There was no difference between the cell counts in the matched controls and incubator (negative control) after 48 hours, but the 1280 J/cm^2^ RL group had significantly lower cell count compared to matched and negative control as assessed with ANOVA with Tukey’s post-hoc analysis. *** denotes p<0.001 and **** denotes p<.0001.


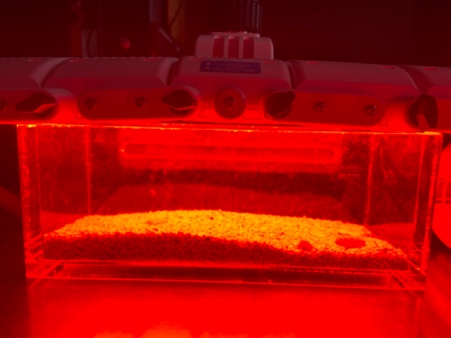


A

B

**Supplemental Figure 2: RL treatment apparatus.** Mice were treated with RL from above and cooled by an attached air-conditioning unit. **(A)** schematic and **(B)** photo of treatment set-up with LED activated.


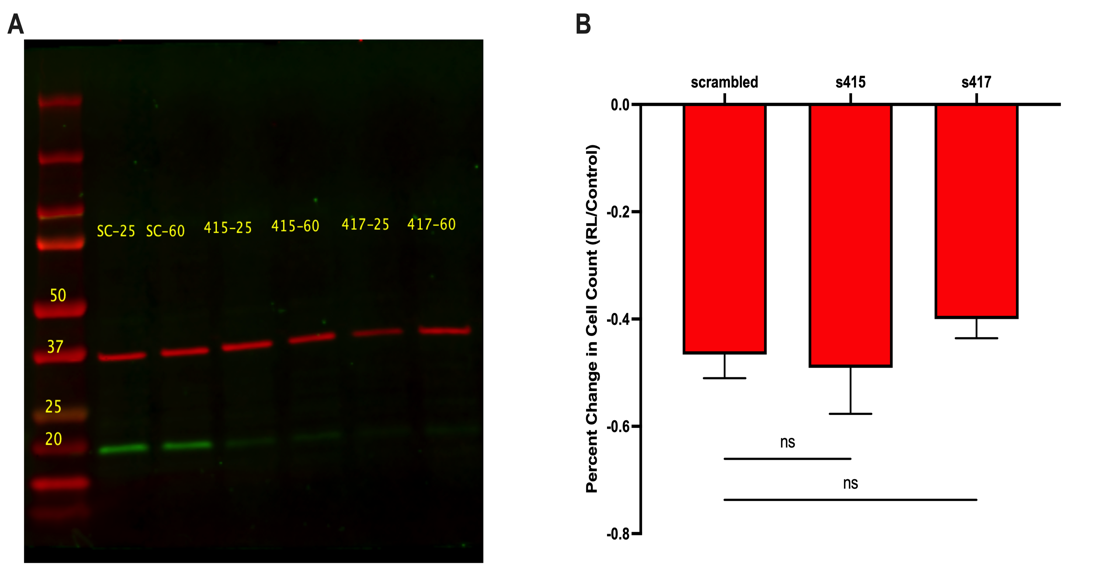


**Supplemental Figure 3. Knockdown of p21 did not restore A375 cell counts following RL.** **(A)** A375 cells were transfected with siRNA SC, s415, or s417 p21 knockdown vectors and replated for experimental protocols. Representative western blot with p21 monoclonal rabbit antibody (1:1000; green) and GAPDH monoclonal mouse antibody (1:10,000; red) for protein form A375 cells transfected with 25 or 60 pmol SC, s415, and s417 p21 knockdown vectors. Molecular weight markers for Bio-Rad dual stain protein ladder are noted. **(B)** 25 pmol SC, s415, and s417 vector treated A375 cells were then irradiated with 640 J/cm^2^ RL. After 48 hours, the cell count was assessed in the RL irradiated and non-irradiated control cells. Bars represent the percent decrease in cell count (RL/Control-1). Percent decrease in cell count between the SC, s415, and s417 vectors were compared using ANOVA (n=5, p<0.05). Abbreviations: SC- scrambled control, SC-25 – 25 pmol of scrambled control vector, SC-60 – 60 pmol of scrambled control vector, s415-25 – 25 pmol of s415 p21 vector, s415-60 – 60 pmol of s415 p21 vector, s417-25 – 25 pmol of s415 p21 vector, s417-60 – 60 pmol of s415 p21 vector, ns – not significant, RL – Red light.


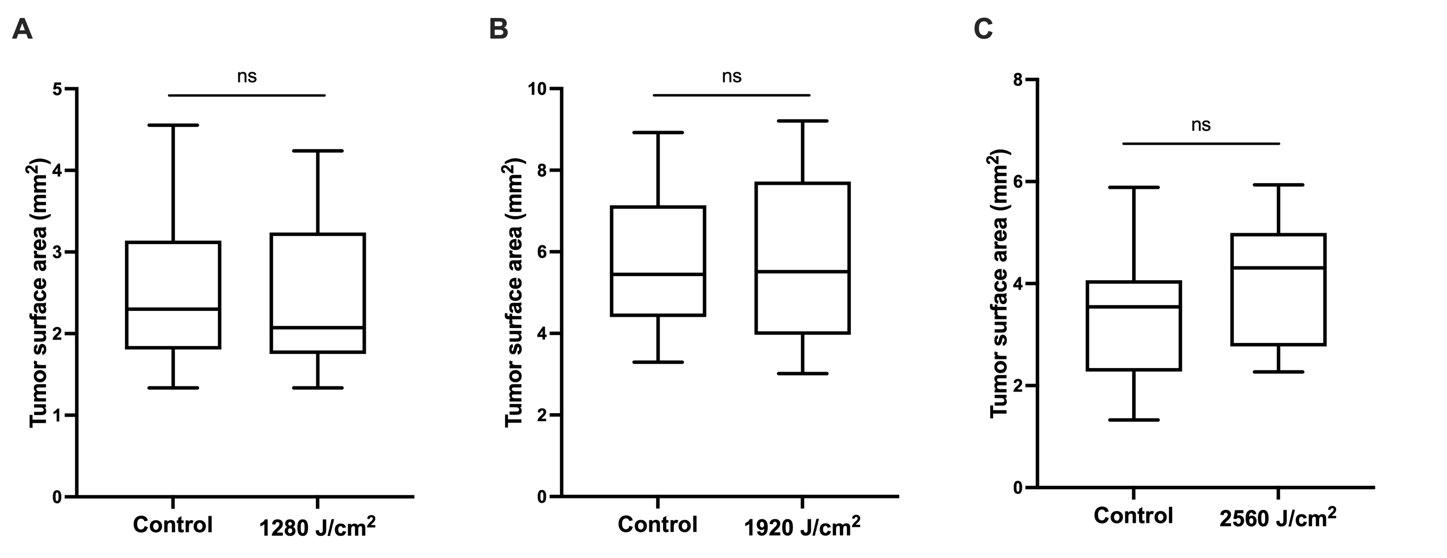


**Supplemental Figure 4. Tumor surface areas were not different between control and RL-treated groups at allocation (day 3).** Mice were injected with 2-3x10^5^ B16F10 cells. On day 3, the tumor dimensions were measured in each mouse before group allocation for **(A)** 1280, **(B)** 1920, and **(C)** 2560 J/cm^2^ regimens. The tumor surface areas were measured, and the mice were sorted by ascending tumor size. Tumor surface area was not significantly different between control and RL-treated groups at allocation (two-tailed T-test, p>0.05). After allocation, groups were assigned to the control or RL treatment regimens. RL irradiation immediately started on day 3. 1280 (n=10), and 1920 (n=10) J/cm^2^ regimens were continued until day 13. 12560 J/cm^2^ (n=12) regimen was continued until day 15. The number of days for 1280, 1920, and 2560 J/cm^2^ regimens depended on humane outcomes (e.g., tumor rupturing or bleeding). All mice in the RL treatment and control group were euthanized when one mouse was observed to have a humane outcome. ns denotes not significant.


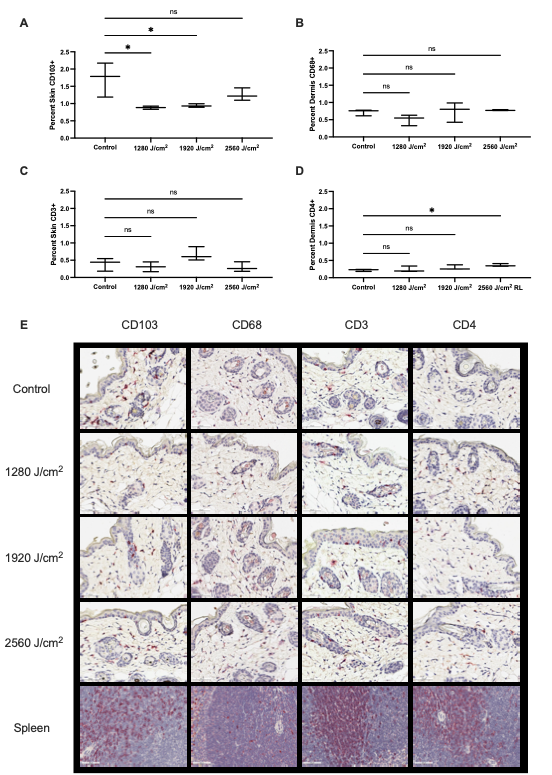


**Supplemental Figure 5: The expression of CD103+ dendritic cells was decreased in normal mouse skin treated with 1280 and 1920 J/cm^2^ RL.** **(A)** Mice without tumors were irradiated with 1280, 1920, or 2560 J/cm^2^ RL for 15 days. Quantification of CD103, **(B)** CD68, **(C)** CD3, and **(D)** CD4 staining in the normal dermis or skin from 1280, 1920, and 2560 J/cm^2^ RL-treated C57BL6 without tumors (n=3, ANOVA, p<0.05). **(E)** Representative IHC for CD103, CD68, CD3, and CD4 positive dermis and skin from C57BL/6 mice without tumors. Spleen positive control tissue is provided. Dunnett's post-hoc testing compared the mean of every RL group to control. Staining was quantified using Indica HALO software compared to total dermal or skin area.* denotes p<0.05
